# Exploration of sedentary behaviour among GPs: a cross-sectional study

**DOI:** 10.3399/BJGPO.2021.0196

**Published:** 2022-03-23

**Authors:** Richard S Mayne, Nigel D Hart, Mark A Tully, Jason J Wilson, Jan C Brønd, Neil Heron

**Affiliations:** 1 School of Medicine, Dentistry and Biomedical Sciences, Queen’s University Belfast, Belfast, UK; 2 Institute of Mental Health Sciences, School of Health Sciences, Ulster University, Newtownabbey, UK; 3 Sport and Exercise Sciences Research Institute, School of Sport, Ulster University, Newtownabbey, UK; 4 Department of Sports Science and Clinical Biomechanics, University of Southern Denmark, Odense, Denmark; 5 School of Medicine, David Weatherall Building, Keele University, Keele, UK

**Keywords:** sedentary behaviour, physical activity, exercise, primary health care, general practice

## Abstract

**Background:**

Sedentary behaviour, which may have increased among GPs due to increasing use of telemedicine, is associated with many illnesses and increased all-cause mortality.

**Aim:**

To explore levels of sedentary behaviour among GPs and General Practice Specialty Trainees (GPSTs).

**Design & setting:**

Sequential, cross-sectional design (initial online sedentary behaviour questionnaire and subsequent thigh-worn accelerometer substudy) of GPs and GPSTs in Northern Ireland.

**Method:**

Self-reported questionnaire data were aggregated and compared with device-measured accelerometry data.

**Results:**

Data from 353 participants (17.7% of GPs and GPSTs in Northern Ireland) revealed doctors in general practice self-reported higher workday sedentary time (10.33 hours, SD 2.97) than those in secondary care (7.9 hours, SD 3.43 [mean difference {MD} 2.43 hours; *P*<0.001]). An active workstation (for example, sit-stand desk), was used by 5.6% of participants in general practice, while 86.0% of those without one would consider using one in future. Active workstation users self-reported lower workday sedentary time (7.88 hours, SD 3.2) than non-users (10.47 hours, SD 2.88 [MD –2.58 hours, *P* = 0.001]). Accelerometer substudy participants underestimated their workday sedentary time by 0.17 hours (95% confidence interval [CI] = –1.86 to 2.20; *P* = 0.865), and non-workday sedentary time by 2.67 hours (95% CI = 0.99 to 4.35; *P* = 0.003). Most GPs (80.7%) reported increased workday sitting time compared to prior to the COVID-19 pandemic, while 87.0% would prefer less workday sitting time.

**Conclusion:**

GPs have high levels of workday sedentary time, which may be detrimental to their health. It is imperative to develop methods to address sedentary behaviour among GPs on workdays, both for their own health and the health of their patients.

## How this fits in

Excessive sedentary behaviour is associated with many adverse health outcomes and increased all-cause mortality, yet little previous research has examined sedentary behaviour among GPs. This study shows that general practice is a highly sedentary occupation, particularly in light of the recent increased use of telemedicine, which may be detrimental to the health of GPs. Most GPs would prefer to spend less time sitting on workdays; in doing so, they could improve both their own health and, potentially, the health of their patients, owing to their ability to effectively counsel patients on healthy lifestyle choices.

## Introduction

Sedentary behaviour is defined as time spent sitting, lying, or reclining, in a state of low-energy expenditure, while awake.^1^ Excessive sedentary behaviour is associated with adverse health outcomes, including type 2 diabetes mellitus, obesity, cardiovascular disease, metabolic syndrome, dementia, certain cancers, mental health issues,^2–4,4^ and increased all-cause mortality.^4–10,6,7,8,9,10^ The World Health Organization, therefore, advises individuals to minimise and break up periods of sedentary behaviour.^11^

Primary care is 'the cornerstone' of the UK NHS, providing over 300 million patient consultations per year.^12^ By virtue of their position in the healthcare system, GPs can provide evidence-based lifestyle guidance to patients, which can play an important role in primary and secondary prevention of many illnesses. GPs who are more physically active are more likely to recommend physical activity to their patients.^13–17,15,16,17^ Patients are more likely to make healthy lifestyle changes if they believe their doctor follows the guidance themselves.^18–20,20^ Reducing sedentary behaviour and increasing physical activity among GPs could, therefore, lead to health benefits for GPs and their patients. This is particularly relevant now that GPs are performing more remote consultations,^21,22^ traditionally performed while sitting down. Core opening hours for general practices are typically around 10 hours every weekday, excluding bank holidays, which means many GPs are in work for most of the time they are awake on a typical workday. It is, therefore, important to investigate current levels of sedentary behaviour among GPs.

The specific objectives of this study were:

- to quantify total daily sedentary time among GPs and GPSTs during a typical workday and non-workday;
- to identify differences in the levels of sedentary behaviour depending on work environment, age, and sex;
- to establish current uptake of ‘active workstations’ such as standing desks; and
- to ascertain if sedentary behaviour has been affected by changes owing to the COVID-19 pandemic.

## Method

### Study design

A cross-sectional study was conducted in accordance with Strengthening the Reporting of Observational studies in Epidemiology (STROBE) guidance,^23^ following a sequential design, incorporating an online questionnaire survey and subsequent accelerometer substudy.

### Stage 1: online questionnaire study

#### Design and distribution

A questionnaire (see Supplementary Appendix S1) was distributed to all GPs and GPSTs throughout Northern Ireland using email mailing lists (to in-hours and out-of-hours GPs and GPSTs, with the support of out-of-hours, training, and continuing professional development providers), and social media. GPs and GPSTs in Northern Ireland have similar working conditions to their contemporaries throughout the rest of the UK. The International Sedentary Assessment Tool (ISAT) was used, which is a multi-item questionnaire developed following a systematic review of sedentary behaviour questionnaires.^24^ Baseline details gathered included age, sex, job role, and working environment. Additional questions explored access to and willingness to use an active workstation, and changes in sitting time since before the COVID-19 pandemic. At the end of the questionnaire, if participants indicated they were now spending more or less time sitting than before the COVID-19 pandemic, they were able to submit a free-text response explaining why. The questionnaire was accessed using a hyperlink to a Microsoft Forms webpage. Participants were recruited voluntarily, with no obligations or rewards for taking part. All participants provided informed consent. The questionnaire was live between 28 August and 24 September 2020.

#### Inclusion and exclusion criteria

Inclusion criteria were being a GP partner, salaried GP, sessional or locum GP, or GPST working in Northern Ireland at the time of the study. Exclusion criteria were answering a question that contradicted the inclusion criteria.

#### Analysis

Responses were reviewed to ensure there were no duplicates. Statistical analyses were conducted using IBM SPSS Statistics (version 25.0). Baseline characteristics were described using mean (SD) for numerical data and counts (%) for categorical data. The distribution of numerical data were assessed visually using histograms and QQ plots. Data were analysed using independent *t*-tests and χ^2^ tests where appropriate. All tests were two-sided with statistical significance set at *P*<0.05.

### Stage 2: accelerometer substudy

#### Recruitment and data collection

Twenty questionnaire responders were recruited to the accelerometer substudy. No sample size calculation was performed, as the primary aim was to gather baseline data. Twenty accelerometers were available to the researchers for concurrent use. Purposive sampling was used to ensure maximum variation based on demographic criteria (age, sex, work pattern, or environment) and self-reported sedentary time. During autumn 2020, participants were posted an Axivity AX3 accelerometer, adhesive waterproof dressings, and instructions. Axivity AX3 accelerometers are valid for accurately identifying sedentary time.^25^ Participants were instructed to wear the accelerometer continuously, on the middle of the thigh, over a 7-day period while completing a contemporaneous sleep and work log. On completion, participants posted back the accelerometer and sleep and/or work log.

#### Inclusion and exclusion criteria

Inclusion criteria were as follows: being a GP partner, salaried GP, sessional or locum GP, or GPST working in general practice in Northern Ireland at the time of the study; having completed the online sedentary behaviour questionnaire; and having consented to being approached for a subsequent accelerometer substudy. Exclusion criteria were as follows: not meeting the inclusion criteria; having a comorbidity that the participant felt would affect sedentary time; being on annual leave during the study; and undertaking contact sports that could damage the accelerometer.

#### Analysis

Accelerometers were programmed to capture triaxial accelerations at 50 Hz with a dynamic range of ±8 g. Details on accelerometer data processing and analysis can be found in a previous study.^26^ For inclusion in the final analysis, accelerometers needed to be worn for a minimum of one valid workday and one valid non-workday. A valid day required a minimum of 600 minutes of wear-time while awake, as required for previous accelerometer studies.^27^ A valid workday required the participant to work at least one clinical session. Accelerometer data were used to determine sedentary time, step count, and time spent during light physical activity (LPA) and moderate-to-vigorous physical activity (MVPA).

## Results

### Online questionnaire

#### Sample characteristics

There were 1999 GPs and GPSTs working in Northern Ireland at the time of the study; 1633 GPs and 366 GPSTs. The online survey was accessed by 353 people, 17.7% of the eligible population. One person answered no questions and three answered no questions apart from number of sessions worked. They were excluded from the analysis.

Summary data of questionnaire participants are included in Table 1. Average age was 39.9 (SD 10.3) years, with 61.6% (*n* = 204) female. GPs comprised 74.2% (*n* = 259), with the rest GPSTs. GPs and GPSTs in general practice at the time of the study comprised 92.0% (*n* = 321), with an average age of 40.7 (SD 10.2) years. The remainder, all GPSTs, with an average age of 32.5 (SD 7.7) years, were working in secondary care settings. GPs reported working an average of 6.09 (SD = 1.75) clinical sessions per week in general practice, while 75.6% of GPSTs were working full-time, with the remainder working part-time.

**Table 1:** Questionnaire survey and accelerometer substudy participant comparisons

Characteristic	Questionnaire survey (*n* = 349)	Accelerometer substudy (*n* = 20)
Age, years*,* mean (SD)	39.9 (10.3)	39.1 (9.7)
Sex, female/male, *n* (%)	204 (61.6) / 127 (38.3)	12 (60.0) / 8 (40.0)
Job role, GP/GPST, *n* (%)	259 (74.2) / 90 (25.8)	16 (80.0) / 4 (20.0)
GP clinical sessions per week, mean (SD)	6.09 (1.75)	6.65 (1.53)
Access to active workstation, no/yes, n (%)	302 (94.4) / 18 (5.6)	16 (80.0) / 4 (20.0)
Average self-reported workday sedentary time, hours, mean (SD)	10.33 (2.97)	9.80 (3.19)
Average self-reported non-workday sedentary time, hours, mean (SD)	4.78 (3.02)	4.38 (2.65)

GPST = general practice specialty trainees. SD = standard deviation.

This data represents staff working in primary care only. Some responders to the questionnaire survey (*n* = 349) did not answer the questions on sex and access to active workstation.

#### Self-reported sedentary time

Overall, participants working in primary care reported more sedentary time on workdays (10.33 hours, SD 2.97, Table 1) than non-workdays (4.78 hours, SD 3.02 [MD 5.55 hours, 95% CI = 5.08 to 6.02, *P*<0.001]) (data not shown). Participants in general practice reported more workday sedentary time (10.33 hours, SD 2.97, Table 1) than those in secondary care (7.9 hours, SD 3.43 [MD 2.43 hours, 95% CI = 1.2 to 3.37, *P*<0.001]). However, participants in general practice reported less sedentary time on non-workdays (4.78 hours, SD 3.02, Table 1), than those in secondary care settings (6.17 hours, SD 3.67 [MD 1.38 hours, 95% CI = 0.17 to 2.60, *P* = 0.025]).

#### Access to active workstations

Summary questionnaire data regarding active workstations is found in Table 2. Among participants in general practice, 5.6% (*n* = 18) reported having access to an active workstation, such as a standing desk, at work. They reported lower workday sedentary time (*P*<0.001) than those who did not have access to an active workstation (7.88 hours, SD 3.20 versus 10.47 hours, SD 2.88). Participants in general practice with active workstations had similar levels of workday sedentary time to participants working in secondary care settings (MD 0.02 hours, 95% CI = –2.10 to 2.06, *P* = 0.985).

**Table 2:** Summary questionnaire data for GPs and GPSTs working in general practice

Category	Do not have active workstation	Have active workstation	Significance
Total, *n (%*)^a^	302 (94.4)	18 (5.6)	—
Sex, female/male, *n (%*)	173 (60.9) / 111 (39.1)	13 (72.2) / 5(27.8)	χ^2^ (1, *n* = 302) = 0.915, *P* = 0.339
Age, years, mean (SD)	40.8 (10.1)	39.0 (10.60)	MD 1.8, 95% CI = –3.19 to 6.87, *P* = 0.472
Overall workday sedentary time, hours, mean (SD)	10.47 (2.88)	7.88 (3.20)	MD 2.58, 95% CI = 1.12 to 4.07, *P* = 0.001
Overall non-workday sedentary time, hours, mean (SD)	4.88 (3.12)	4.36 (2.67)	MD 0.52, 95% CI = –0.50 to 1.11, *P* = 0.304

GPST = general practice specialty trainee. MD = mean difference. SD = standard deviation.

^a^One responder did not answer this question.

#### Attitudes regarding active workstations

Among participants in general practice without active workstations, 86.0% (*n* = 253) would consider using one in future (data not shown). Participants who would consider using an active workstation were younger (40.2 years, SD = 9.7 versus 45.3 years, SD 12.1; *P* = 0.019) than those who would not.

#### Attitudes regarding sedentary behaviour

Among participants in general practice, 87.0% (*n* = 274) reported they would prefer less time sitting, 11.9% (*n* = 38) would prefer the same time sitting, and 1.1% (*n* = 3) would prefer more time sitting on a typical workday (data not shown). Those who would prefer less time sitting had more (*P*<0.001) workday sedentary time (10.68 hours, SD 2.70) than those who would prefer the same time sitting (7.93 hours, SD 3.45).

#### Changes in sedentary behaviour owing to the COVID-19 pandemic

Among participants in general practice, 80.7% (*n* = 255) reported spending more time sitting, 3.9% (*n* = 44) the same time sitting, and 5.4% (*n* = 17) less time sitting at work than before the COVID-19 pandemic (data not shown). Remote consulting was cited in the free-text responses of 94.5% (*n* =241) of the 255 participants who reported more time sitting.

### Accelerometer substudy

#### Sample demographics

Of the 353 participants who accessed the initial online questionnaire survey, 195 consented to being approached for the subsequent accelerometer substudy. Forty-six survey participants were invited to participate in the accelerometer substudy. The accelerometer substudy invitation email received no response from 17 recipients. Of the 29 survey participants who responded to the invitation email, 20 agreed to participate. Nine did not meet the inclusion criteria: four were on annual leave; two declined; two participated in contact sports; and one had a comorbidity. Table 1 compares accelerometer substudy participants with questionnaire survey participants.

#### Data capture and analysis

All accelerometers and sleep and work logs were returned to the investigators. Not all participants wore accelerometers during the study period: two forgot to wear the device; and one was unable to affix the device to their thigh. Therefore, 17 participants provided usable accelerometer data to analyse.

#### Comparison of accelerometer and self-reported data

Two participants who wore the accelerometer were excluded from the analysis. They did not work in general practice because of illness during the study. Objective accelerometer data were compared with subjective, self-reported data for the remaining 15 participants, and summarised in Table 3. Average self-reported workday sedentary time was 9.83 (SD 3.45) hours. Their average accelerometry-measured workday sedentary time was 10.00 (SD 1.69) hours, showing they had slightly underestimated their overall workday sedentary time by 0.17 hours (*P* = 0.865). Average non-workday self-reported sedentary time was 4.53 (SD 2.55) hours. Their average accelerometry-measured overall non-workday sedentary time was 7.20 (SD 1.88) hours, showing they had significantly underestimated their overall non-workday sedentary time by 2.67 hours (*P* = 0.003).

**Table 3:** Comparison of accelerometry-measured and self-reported sedentary time

Category	Self-reported (*n* = 15),mean (SD)	Accelerometry-measured (*n* = 15),mean (SD)	Mean difference, 95% CI, *P* value
Workday sedentary time, hours	9.83 (3.45)	10.00 (1.69)	0.17, –1.86 to 2.20, 0.865
Non-workday sedentary time, hours	4.53 (2.55)	7.20 (1.88)	2.67, 0.99 to 4.35, 0.003

SD = standard deviation.

#### Active workstations

Accelerometery-measured data regarding active workstations is summarised in Table 4. Participants with active workstations (*n* = 4) had less workday sedentary time (*P*<0.001) than those without active workstations (*n* = 11) (7.57 hours, SD 0.56 versus 10.88 hours, SD 0.81). They also had more (*P*<0.001) workday standing time (5.81 hours, SD 1.39 versus 2.88 hours, SD 0.79). There was no significant difference in average workday LPA, MVPA, and step counts between participants with and without active workstations.

**Table 4:** Accelerometer substudy comparison regarding active workstations

Category	No access to active workstation (*n* = 11),mean (SD)	Access to active workstation (*n* = 4),mean (SD)	Mean difference, 95% CI, *P* value
Workday sedentary time, hours	10.88 (0.81)	7.57 (0.56)	3.31, 2.36 to 4.28, <0.001
Workday standing time, hours	2.88 (0.79)	5.81 (1.39)	2.93, 1.71 to 4.14, <0.001
Workday LPA, hours	3.28 (0.79)	3.53 (1.23)	0.25, –1.39 to 0.91, 0.659
Workday MVPA, hours	0.37 (0.34)	0.34 (0.20)	0.03, –0.36 to 0.43, 0.852
Workday step count, steps	5331.09 (3096.62)	5145.19 (862.29)	MD 185.90, –3651.33 to 3279.53, 0.910

LPA = light physical activity. MVPA = moderate-to-vigorous physical activity.

#### Workdays versus non-workdays

Comparison of workdays and non-workdays is summarised in Table 5. Sedentary time was higher (10.00 hours, SD 1.69 versus 7.20 hours, SD 1.88; *P*<0.001) on workdays than non-workdays. LPA (3.36 hours, SD 0.86 versus 4.26 hours, SD 1.26*; P* = 0.030), MVPA (0.36 hours, SD 0.29 versus 1.02 hours, SD 0.41; *P*<0.001) and step counts (5281.51 steps, SD 2690.17 versus 10 890.89 steps, SD 4063.56*; P*<0.001) were lower on workdays than non-workdays. There was no significant difference in standing time on workdays and non-workdays.

**Table 5:** Accelerometer substudy comparison regarding workdays versus non-workdays

Category	Workday (*n* = 15),mean (SD)	Non-workday (*n* = 15),mean (SD)	Mean difference, 95% CI, *P* value
Overall sedentary time, hours	10.00 (1.69)	7.20 (1.88)	2.80, 1.46 to 4.14, <0.001
Overall standing time, hours	3.66 (1.58)	4.18 (1.32)	0.52, –0.57 to 1.61, 0.336
LPA, hours	3.36 (0.86)	4.26 (1.26)	0.90, 0.09 to 1.71, 0.030
MVPA, hours	0.36 (0.29)	1.02 (0.41)	0.66, 0.39 to 0.93, <0.001
Step count, steps	5281.51 (2690.17)	10 890.89 (4063.56)	5609.38, 3597.21 to 7621.55, <0.001

LPA = light physical activity. MVPA = moderate-to-vigorous physical activity.

## Discussion

### Summary

To the authors’ knowledge, this is the first study to specifically examine levels of sedentary behaviour among GPs. Participants had significantly more sedentary time on workdays compared with non-workdays. Those in general practice with active workstations had similar levels of workday sedentary time to those in secondary care. Participants in general practice without active workstations had significantly higher levels of workday sedentary time than those with active workstations, or those in secondary care. Participants with active workstations primarily replaced sedentary time with standing time. GPs now report having higher levels of workday sedentary time than before the COVID-19 pandemic. Most would prefer less sedentary time. Despite only a small minority of GPs currently having access to active workstations, a large majority, particularly those younger in age groups, would consider using one in future.

### Strengths and limitations

An online survey was less onerous for participants than a paper-based, postal survey. Multi-item questionnaires with relatively short recall periods are more reliable than single-item questions and longer-recall periods.^26^ Thigh-worn accelerometers are highly accurate for identifying sedentary behaviour.^28^ Using accelerometers with an accompanying sleep and work log among a smaller, purposive sample of participants allowed comparison between subjectively and objectively reported sedentary time and between workdays and non-workdays.

The questionnaire response rate of 17.7% is similar to previous online surveys among GPs.^28–30,30^ Higher response rates have been obtained by postal surveys;^30,31^ however, this may have caused increased hassle for participants, particularly in light of concerns regarding higher workload during the COVID-19 pandemic.^32^ COVID-19 restrictions prevented face-to-face recruitment, which may also have improved the overall response rate. Demographic data of participants appears to be comparable with published governmental data of GPs in Northern Ireland;^33^ however, the relatively low response rate means that participants may not have been truly representative of all GPs and GPSTs in Northern Ireland at the time of the study. Thigh-worn accelerometers are unable to detect upper-body movement, so if a participant was sitting or lying while performing exercise involving the trunk or arms, this may incorrectly have been recorded as sedentary behaviour. Participants in the accelerometer substudy may have modified their behaviour while they were wearing the device; however, the significance of this is uncertain and is shared with other studies using similar devices for objective measurements.^34,35^

### Comparison with existing literature

A recent systematic review, conducted by the authors, identified two previous studies reporting levels of sedentary behaviour among GPs.^36^ Keohane *et al* examined GP trainees and GP trainers in Ireland in 2018, when 60% reported spending in excess of 7 hours, 24% between 4 and 7 hours, and 16% ≤4 hours sitting each day.^29^ Suija *et al* examined female GPs in Estonia in 2009, reporting mean daily sitting time of 6 hours and 36 minutes, with 56% sitting for >6 hours per day.^28^ Both studies primarily examined physical activity using the International Physical Activity Questionnaire (IPAQ),^28,29^ where participants were asked how much time they usually spend sitting on an average weekday.^37^ This may have underestimated sedentary behaviour, which includes when an individual is awake in a lying or reclining posture.^1^ Both studies also took place before the COVID-19 pandemic, which has resulted in significant, potentially longstanding changes to general practice.^38^ The findings in this study reflect the increased use of remote consultations brought about by the COVID-19 pandemic.^22^

This study shows that most GPs currently have >10 hours of total sedentary time over the course of each workday, which is more than previously reported and similar to workers in the education, telecom, and service industries.^39^ This is a concerning finding given the established dose-response relationship between sedentary time and mortality.^4–10,6,7,8,9,10^ Mortality risk has been shown to increase gradually between 7 and 9 hours of average daily sedentary time, with a further increase >9 hours.^8^ For participants working in secondary care settings, or in general practice with active workstations, their average overall workday sedentary time of <8 hours could potentially make them less likely to be affected by the adverse health outcomes associated with excessive sedentary behaviour. Active workstations allow the user to alternate between sitting and standing, which has been shown to reduce postprandial glycaemia excursion, blood pressure, and back pain.^40^ The greater disparity in self-estimated versus accelerometry-measured sedentary time on non-workdays, compared with workdays, aligns with previous studies finding self-reported sedentary time to be more accurate on a workday than on a non-workday.^41–43,43^ This may be because workdays follow a more reliable, predictable structure and routine than non-workdays, which may be less structured and more variable.

### Implications for research and practice

This study demonstrates that doctors working in general practice typically have high levels of sedentary time on workdays, with much less on non-workdays. It is, therefore, important to consider ways of reducing workday sedentary time among GPs, given the negative health effects of excessive sedentariness and the role of GPs in counselling patients about healthy lifestyles. If GPs were able to find solutions to reduce their own workday sedentary behaviour, they could share these with patients when discussing how patients could reduce their sedentary behaviour both in and out of the workplace. One potential approach is the use of active workstations, which are already being used by a minority of GPs. Although active workstation users had less workday sedentary time than non-users, their sedentary time was primarily replaced by static standing time. Multi-component interventions to reduce workday sedentary behaviour and increase physical activity may be more successful. Future research should assess whether levels of sedentary time and physical activity among GPs changes with the easing of restrictions related to the COVID-19 pandemic and the adoption of new technologies. It would also be relevant to assess sedentary behaviour and physical activity throughout the primary care multidisciplinary team. Qualitative research focusing on the enablers and barriers to GPs reducing their workday sedentary time would shed more light on the acceptability and feasibility of future interventions in this area.
